# Removal of Sulfide Ions from Kraft Washing Effluents by Photocatalysis with N and Fe Codoped Carbon Dots

**DOI:** 10.3390/polym15030679

**Published:** 2023-01-29

**Authors:** Hao Luo, Hao Liu, Chengwu Sun

**Affiliations:** 1 State Key Laboratory of Pulp and Paper Engineering, South China University of Technology (SCUT), Guangzhou 510640, China; 2 Bengbu-SCUT Research Center for Advanced Manufacturing of Biomaterials, Bengbu 233010, China

**Keywords:** N and Fe codoped carbon dots (N,Fe-CDs), sulfide ions (S^2−^), photocatalysis, kraft washing effluents (KWE)

## Abstract

N and Fe codoped carbon dots (N,Fe-CDs) were fabricated from citric acid, L-glutamic acid and ferric chloride via a hydrothermal method for the photocatalytic removal of S^2−^ from kraft washing effluents (KWE). The N,Fe-CDs were fluorescent nanoparticles (average size of 3.18 nm) and catalyzed the oxidation of S^2−^ following a first-order kinetic model with an activation energy of 33.77 kJ/mol. The N,Fe-CDs tolerated elevated temperatures as high as 80 °C without catalyst deactivation. The N,Fe-CDs catalysts were reusable for at least four cycles, preserving over 90% of the activity. In the treatment of KWE from the kraft pulping of eucalyptus, the concentration of S^2−^ was decreased by the N,Fe-CDs from 1.19 to 0.41 mmol/L in 6 h. Consequently, near complete remediation was obtained in 24 h. In addition, half of the chemical oxygen demand was removed after treatment with 500 mg/L of the N,Fe-CDs. In addition, the present photocatalyst was safe within a concentration of 200 mg/L, as indicated by the acetylcholinesterase inhibition test. Our findings may help develop a cleaner production process for kraft brownstock washing.

## 1. Introduction

Kraft cooking is currently the dominant chemical pulping method contributing to about 90% of the global production of brownstock pulp [1]. In both batch and continuous kraft processes, wood chips are delignified at a given H factor with white liquor, composed of NaOH and Na_2_S, to reach a desired kappa number [2]. After cooking, brownstock slurry is delivered into a blow tank and subsequently sent to pulp washers [3]. About 90% of spent cooking liquor, or so-called black liquor, separated from the washing process is subjected to evaporators and boilers for the recovery of heat and chemicals [4]. The rest is primarily diluted with washing water and pumped to an effluent treatment system (ETS) [5]. In the kraft pulping of non-woody biomass, the extraction percentage of black liquor is usually lower than 80% [6], which means 20% of the spent chemicals enter the subsequent units. Sulfide ions (S^2−^, or HS^−^ under alkaline pH) are one of the major hazardous chemicals in kraft washing effluents (KWE) [7]. They impact the ETS by declining the performance of activated sludge due to their high oxygen demand and strong biological toxicity [8]. There is urgent need to remediate S^2−^ prior to the active sludge stages.

A routine solution is to add microbial promoters, e.g., sulfur-oxidizing bacteria, together with a bio-energizer to restore the efficiency of activated sludge to a normal level [9]. However, this approach usually takes a few days, not to mention the significant increase in operation costs. Conventional chemical sulfide oxidation employs ClO_2_ or Fenton’s reagents [10,11]. The former technology requires additional equipment for the generation of ClO_2_ [10]. The latter requires acidic conditions and generates a considerable amount of ferric sludge [11]. It is desired to develop biosourced recyclable catalysts for the green remediation of S^2−^ in KWE.

Carbon dots (CDs) are a new type of photocatalysts with a small particle size, high chemical stability and excellent catalytic performance [12]. CDs have been widely studied in pollution control and waste valorization since they were first synthesized in 2004 [13]. In general, CDs particles consist of carbon cores with outer layers rich in functional groups (-COOH, -OH, -NH_2_) as well as codoped metal and/or non-metal atoms if designed as such [14]. Light irradiation could effectively excite the catalytic activity of CDs by promoting the migration of electrons, which finally accelerates the degradation of pollutants [15]. However, to date, CDs have not been studied for sulfide removal from industrial effluents. Instead, there have been many reports on the quantification of S^2−^ and sulfide compounds using CDs as detection probes [16,17,18]. For instance, CD/AgI hybrid probes were fabricated using citric acid as a precursor, enabling the measurement of the S^2−^ concentration in a range of 1~10 μM. A minimum detection limit of 0.48 μM was obtained suggesting a very high sensitivity [19]. In another report, dual-emission fluorescent probes were prepared by doping copper nanoclusters in CDs for the determination of trace sulfides with an accuracy of 1 ppm [20]. Novel stable CD/MnO_2_ nanosheets were developed by Liu et al., which could serve as a turn-on fluorescent sulfide probe [21].

The objective of this work is to fabricate N,Fe-CDs specifically for the removal of S^2−^ from KWE. The doping of Fe aims to enhance the local electron density of the material matrix and promote intermolecular electron transfer [22]. Doping of N facilitates the entry of photogenerated electrons into the conduction band [23]. To the best of our knowledge, this article provides the first detailed assessment of CDs for sulfide remediation in pulping effluents. The catalytic performance of the N,Fe-CDs was evaluated. A preliminary study of safety was performed as well.

## 2. Materials and Methods

### 2.1. Chemicals

Elemental iodine, sodium thiosulfate, ferric chloride, soluble starch, L-glutamic acid and citric acid were purchased from Macklin Chemical Reagent Co., Ltd. (Shanghai, China). Other chemicals were of analytical grade and were supplied by Guangzhou Chemical Reagent Factory (Guangzhou, China).

### 2.2. Synthesis of N,Fe-CDs

**Figure 1:**
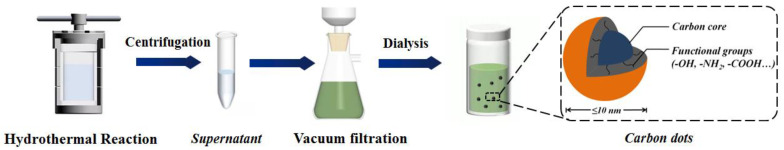
Fabrication diagram of N,Fe-CDs.

The fabrication of N,Fe-CDs is illustrated in Figure 1. A total of 2.0 g of citric acid, 1.5 g of L-glutamic acid and 1.0 g of ferric chloride were dissolved sequentially in 70 mL of distilled water at 25 °C. The solution was transferred to a JGF-100 high pressure reactor (Jiean, Shanghai, China) in which a hydrothermal reaction was carried out at 200 °C for 4 h. Then, the reaction mixtures were cooled and centrifuged (5500 rpm, 25 °C) for 20 min to remove precipitates. The supernatant was vacuum-filtered through a 0.22 μm membrane. It was finally dialyzed against distilled water in a cellulose acetate dialysis tubing (MWCO 500 Da) for 48 h.

### 2.3. Kraft Washing Effluent (KWE)

Eucalyptus wood chips were cooked in a horizontal rotary digester (KRK 2611, Japan) under the following conditions: effective alkali charge of 30% (on basis of NaOH), sulfidity of 15%, solid-to-liquid ratio of 1:4, cooking temperature of 170 °C and total H factor of 1555. After kraft cooking, the spent black liquor was separated with a 200 mesh nylon sieve bag. The solids were subsequently washed with 2 L of deionized water filtered through the nylon bag to produce the kraft washing effluent (KWE). The pH of the KWE was 12.7 ± 0.1, and the concentration of S^2−^ was 1.19 ± 0.10 mmol/L.

### 2.4. Characterization of N,Fe-CDs

UV-Vis spectra of the N,Fe-CDs were recorded using a UV-Vis 1900 spectrophotometer (Shimadzu, Kyoto, Japan). Fluorescence emission and excitation spectra were taken on a FluoroMax-4 fluorescence spectrometer (Horiba, Kyoto, Japan). The excitation wavelengths were 280, 300, 320, 340, 360, 380, 400 and 420 nm. Both excitation and emission slits were 5 nm. The N,Fe-CDs were also characterized using FT-IR spectroscopy on a Tensor spectrometer (Bruker Optics, Karlsruhe, Germany). All dried samples were diluted with KBr and pressed into tablets. An X-ray photoelectron spectroscopic (XPS) study was performed on a Kratos AXIS Ultra DLD spectrometer (Shimadzu, Tokyo, Japan).

The particle size distribution of the N,Fe-CDs was measured using a Nano Zetasizer (Malvern, Malvern, UK). For the transmission electron microscopy (TEM) examination, the N,Fe-CDs were deposited on a piece of carbon-coated copper grid and air-dried. The morphology was scanned on a JEM-2100F microscope (JEOL, Tokyo, Japan). Tapping mode atomic force microscopy of the N,Fe-CDs was carried out on a Multimode-8 scanning probe microscope (Bruker, Karlsruhe, Germany). Samples were prepared by depositing dilute N,Fe-CDs solution on fresh mica, followed with drying using N_2_. All data were processed with the Nanoscope analysis software.

### 2.5. Catalytic Oxidation of S^2−^ by N,Fe-CDs

Na_2_S solutions (30~90 mg/L) were prepared using dissolved oxygen-free water to minimize the auto-oxidation of S^2−^. A total of 20~80 mg of the N,Fe-CDs was added into 200 mL of Na_2_S solution in a photoreaction vessel at a certain temperature (20~80 °C) and under light irradiation. To record the time-dependent catalytic oxidation of S^2−^ by the N,Fe-CDs, the reaction mixtures were pumped from the photoreaction vessel into a 10 mm quartz flow cuvette (Figure S1). Real-time variation curves of absorbance values at λ_230nm_ (S^2−^) and λ_215nm_ (S_2_O_3_^2−^) were recorded and denoted as A_230_ and A_215_. Molar concentrations of S^2−^ and S_2_O_3_^2−^ were derived from A_230_ and A_215_ according to Lambert–Beer law. Their respective molar absorption coefficients were 2.28 × 10^3^ and 2.22 × 10^3^ L/mol/cm. Reaction rate constants (k) were obtained by simulating the kinetics data measured under varied temperature and light conditions, following Equation (1).

lnC_0_−lnC_A_ = kt (1)

where C_0_ and C_A_ were concentrations of S^2−^ at the starting time and time t, respectively.

For evaluation of the recyclability of catalysts, 60 mg of N,Fe-CDs were added to the reaction vessel with 200 mL of the Na_2_S solution (50 mg/L). The reaction was maintained at 25 °C for 2 h, monitored with the flowing spectroscopic method as described above. The reactants were dialyzed against water in a cellulose acetate dialysis tubing at the end of first run reaction and subsequently subjected to 200 mL of fresh Na_2_S solution (50 mg/L) for the next run test. A total of six run cycles were tested. The concentration of S^2−^ was determined every 6 min in each cycle.

### 2.6. Catalytic Oxidation of S^2−^ in KWE by N,Fe-CDs

The applicability of photocatalytic oxidation was studied by mixing 10 mg of N,Fe-CDs in 200 mL of the KWE. The treatment was carried out at 25 °C, at 2000 Lux (white LED), for 12 h. Water was used as reference. The concentrations of S^2−^ and S_2_O_3_^2−^ were measured using the titration method because the deep-colored KWE could not be precisely quantified through the direct measurement of UV absorbance [24]. In flask A, 100 mL of the KWE after treatment with N,Fe-CDs was mixed with 50 mL of I_2_ standard solution (0.1 mol/L) and one drop of soluble starch solution (1 wt%). The mixture was titrated with a standard Na_2_S_2_O_3_ solution (0.05 mol/L) until the blue color disappeared. In flask B, 100 mL of the KWE which had undergone treatment with N,Fe-CDs was precipitated by ZnCl_2_. The reactants were centrifuged at 5500 rpm for 10 min to remove solids. The supernatant obtained was mixed with one drop of starch indicator (0.5 wt%). Standard I_2_ solution (0.05 mol/L) was used to titrate the mixture solution until it turned blue.. The concentration of S_2_O_3_^2−^ and S^2−^ were deduced from Equations (2) and (3), respectively.

S^2−^ + I_2_→2I^−^ + S

S_2_O_3_^2−^ + 4I_2_ + 5H_2_O→2SO_4_^2−^ + 8I^−^ + 10H^+^

(2) $$\mathrm{In}\;\mathrm{flask}\;B,\;C\left(S_{2}O_{3}^{2-}\right)=\frac{C_{B}\left(I_{2}\right)\times V_{B}\left(I_{2}\right)}{4V_{w}}$$

where, C_B_ (I_2_) was 0.05 mol/L; V_B_ (I_2_) was the titration volume, L; and V_w_, the volume of ZnCl_2−_treated KWE, was 0.1 L.

(3) $$\mathrm{In}\;\mathrm{flask}\;A,\;C\left(S^{2-}\right)=\frac{C\left(I_{2}\right)\times V\left(I_{2}\right)-4C_{A}\left(S_{2}O_{3}^{2-}\right)\times V_{A}\left(S_{2}O_{3}^{2-}\right)-C_{B}\left(I_{2}\right)\times V_{B}\left(I_{2}\right)}{V_{w}}$$

where C_A_(S_2_O_3_^2−^) was 0.05 mol/L; V_A_(S_2_O_3_^2−^) was the titration volume, L; and V_w_ was the KWE volume, L.

The determination of the SO_4_^2−^ ions was performed by precipitating the KWE with BaCl_2_ solution followed by the collection, drying and weighing of the generated BaSO_4_. The concentration of SO_4_^2−^ was calculated according to Equation (4).

(4) $$C\left({\mathrm{SO}}_{4}^{2-}\right)=\frac{m\left({\mathrm{BaSO}}_{4}\right)}{233.39\times V_{w}}$$

where m represents mass, g; and 223.39 is the relative molecular mass of BaSO_4_, g/mol.

The chemical oxygen demand (COD) of the KWE was measured using a COD determination kit (Huaikai, Guangzhou, China) on the basis of the potassium dichromate method [25]. Potassium hydrogen phthalate was used as the standard for COD determination. The absorbance at 420 nm (A_420_) was measured and the COD was calculated according to Equation (5).

(5) $$A_{420}=-0.0033\times \mathrm{COD}+0.5853,{\;R}^{2}=0.9983,\;n=7$$

### 2.7. Safety Assessment of N,Fe-CDs

Acetylcholinesterase (AchE) inhibition by the N,Fe-CDs was quantitatively determined using a commercial assay kit (HPER Scientific Instrument Co., Ltd., Guangzhou, China) [26]. In a 4-mL cuvette, 0.14 mL of phosphate buffer (20 mM, pH 7.6), 0.05 mL of acetylthiocholine iodide (5 mM) and 0.01 mL of the N,Fe-CDs solution (water as reference) were pipetted sequentially. The mixture was incubated at 38 °C for 10 min. At the end of reaction, 1.8 mL of 5,5’-dithiobis-2-nitrobenzoic acid/phosphate ethanol reagent was added. The change in absorbance at 412 nm in 3 min (ΔA_412_) was recorded from which the relative activity of AchE (RA, %) was reported according to Equation (6).

(6) $$\mathrm{RA}=\frac{\Delta A_{412,\mathrm{Sample}}}{\Delta A_{412,\mathrm{Water}}}\times 100\%$$

## 3. Results

### 3.1. Characterization of N,Fe-CDs

**Figure 2:**
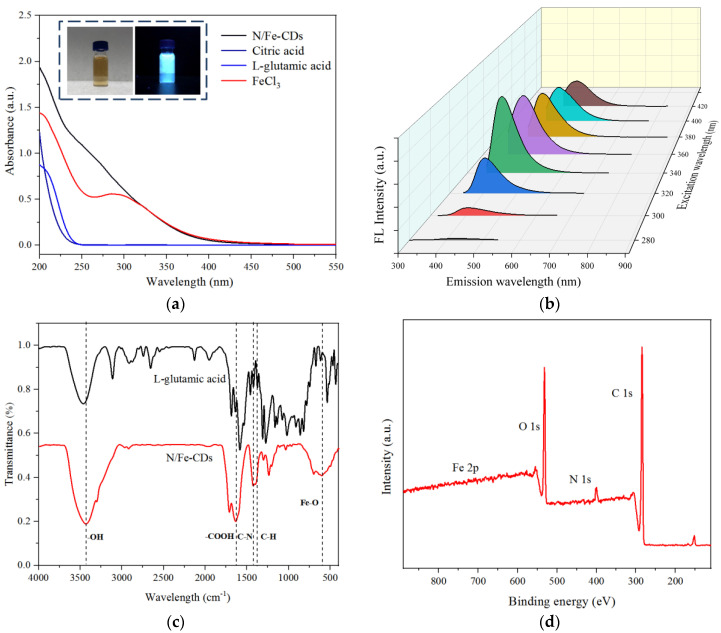
Spectral characteristics of N,Fe-CDs, (**a**) UV-Vis spectra, (**b**) Fluorescence spectra, (**c**) FI-IR spectra, and (**d**) XPS spectra.

The prepared N,Fe-CDs solution was apparently brownish-yellow colored having an absorption spectrum covering the UV region (Figure 2a). No specific peak at around 260 nm was found, suggesting Fe^3+^ was not abundantly bound or perhaps in a chelated state. Fluorescence spectra show the N,Fe-CDs were more readily excited by long wavelength UV lights (340~420 nm) than short ones (280~340 nm) to produce fluorescence. A maximum emission wavelength (E_m_) of 410 nm was obtained when the excitation wavelength (E_x_) was 340 nm (Figure 2b). The previously reported N,Fe-CDs by Wu et al. [27] were derived from ethanediamine and citric acid and had an E_m_ at 450 nm after being excited by 360 nm UV light. Their longer wavelength fluorescence was probably due to the structure of the precursors. On the other hand, fluorescence spectral examinations show the present N,Fe-CDs did not precipitate S^2−^ through the formation of Fe_2_S_3_ because the excited fluorescence produced by the N,Fe-CDs was not influenced by the presence of S^2−^ (0.05~10 mmol/L, Figure S2).

FT-IR spectra showed the N,Fe-CDs had strong transmittance peaks at 1750, 3460 and 1346 cm^−1^ (Figure 2c), corresponding to −COOH, −OH and C−H stretching vibrations, respectively. Bands at around 640 cm^−1^ corresponded to the stretching vibration of Fe−O [28]. The peaks at 1452 cm^−1^ and 1161 cm^−1^ were attributed to the N−H bending vibration and C−N stretching vibration, respectively. Both were present in L-glutamic acid but disappeared in the N,Fe-CDs, showing the amino groups were destroyed in the hydrothermal reaction.

The XPS spectra verified the incorporation of N and Fe in the N,Fe-CDs (Figure 2d). The contents of C, O, N and Fe were 80.5%, 15.7%, 3.8% and 0.1%, respectively. Peak fitting of C spectra resulted in three peaks attributed to C−C (284.7 eV), C−OR (285.8 eV) and C=O (287.7 eV, Figure S3). N elements mostly exist in (398.2 eV) a pyridine-like structure, consistent with the FT-IR results. The binding amount of Fe was quite low. The XPS peaks of Fe included Fe 2p 3/2 (706.75 eV), Fe 2p1/2 (707.91 eV), Fe 2p3/2 (710.96 eV) and Fe 2p1/2 (711.55 eV), respectively (Figure S3). Doping of Fe ions could promote the local electron density of nanoparticles and thereby enhance the intermolecular electron transfer for catalytic activity [29].

**Figure 3:**
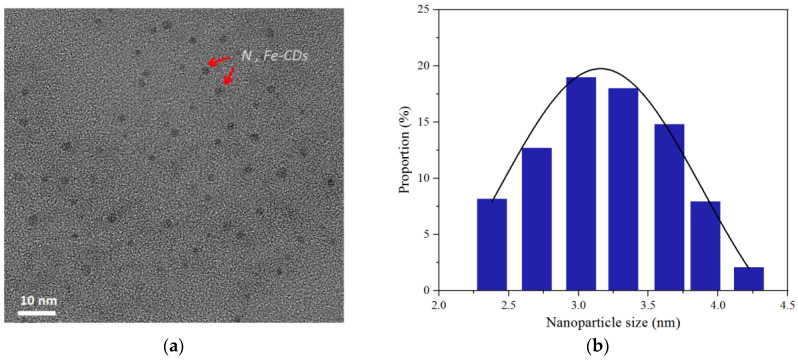

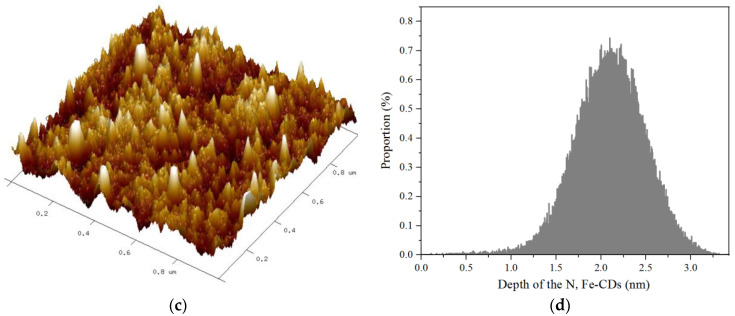
Morphology and size distribution of N,Fe-CDs, (**a**) TEM morphology, (**b**) Size distribution by zetasizer, (**c**) 3D AFM morphology, and (**d**) Size distribution by AFM.

TEM shows the N,Fe-CDs granular nanoparticles dispersed uniformly in aqueous solution without obvious agglomeration (Figure 3a). The particle size of the N,Fe-CDs was 1.5~2.5 nm according to the TEM images which was smaller than the average data, 3.18 nm, measured by zetasizer (Figure 3b). Additional information provided by zetasizer was that all the N,Fe-CDs had size dimensions within a range of 2~4.5 nm. Those particles with size of 2.7~3.65 nm accounted for 65% of the total. AFM offered 3D images of the N,Fe-CDs deposited on mica and the average vertical size of the N,Fe-CDs was 2.3 nm. Data from microscopic measurements lower than the results by zetasizer could be explained by the drying treatment in sample preparation. Our N,Fe-CDs have an average particle size close to the amino-acid-based CDs (3.19 nm) prepared by Pandit et al. [30].

### 3.2. Catalytic Properties of N,Fe-CDs for Oxidation of S^2−^

**Figure 4:**
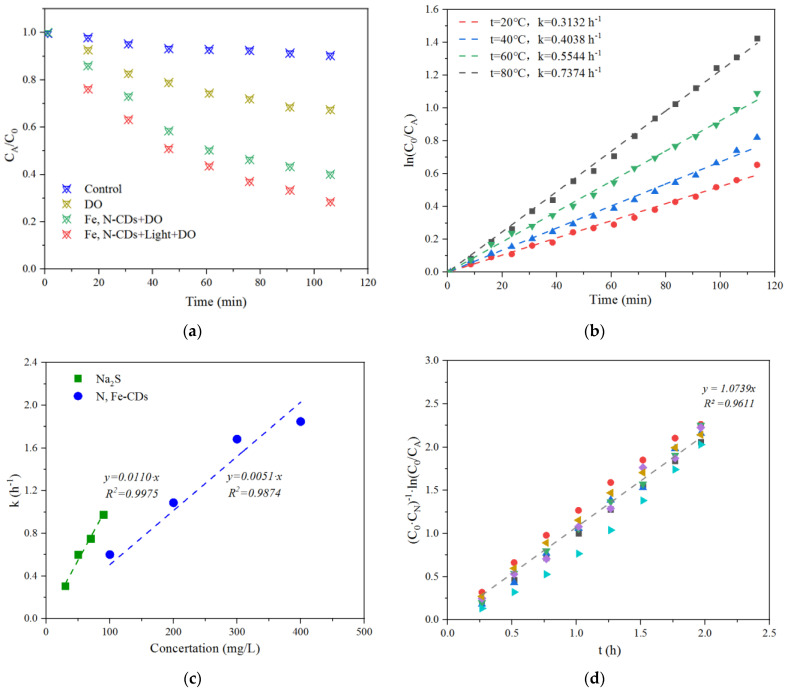
Kinetics of S^2−^ oxidation catalyzed by Fe,N-CDs. (**a**) Time-dependent oxidation of S^2−^ by 100 mg/L Fe,N-CDs with light and DO at 25 °C. (**b**) Kinetic curves at varied temperatures. (**c**) Correlation of k with concentrations of S^2−^ and N,Fe-CDs. (**d**) Fitting of data in Figure 4c with Equation (9).

The catalytic oxidation of S^2−^ by the N,Fe-CDs was kinetically measured under varied conditions (Figure 4). S^2−^ was stable in the absence of dissolved oxygen (DO). In the presence of 6 mg/L of DO, S^2−^ was oxidized slowly. Its content was decreased by 19.4% in 2 h. The N,Fe-CDs accelerated the oxidation even in darkness, eliminating 55.4% of S^2−^ in 2 h. Under white LED irradiation, the N,Fe-CDs were activated and remediated 67.8% of S^2−^ (Figure 4a). Obviously efficient catalysis by the N,Fe-CDs was dependent on O_2_ and light. In a previous report, N,Fe-CDs derived from diethylenetriamine, pentaacetic acid and Fe(NO)_3_ showed peroxidase-like properties, depending on the participation of H_2_O_2_ for the formation of reactive oxygen species [31]. The present catalyst did not require H_2_O_2_ but utilized O_2_ for S^2−^ oxidation.

Kinetic curves of S^2−^ oxidation catalyzed by 100 mg/L of the N,Fe-CDs at varied temperatures are shown in Figure 4b. All curves could be fitted well with the first-order kinetic model (all R^2^ > 0.97). The values of constant k were increased with elevation of temperatures, i.e., from 20 to 80 °C. A total of 75.6% of S^2−^ was oxidized at 80 °C after 120 min. Cui et al. have developed CDs doped with N and S for the probing of Fe^3+^. They found 25 °C was a preferred working temperature, and fluorescence intensity decreased linearly with temperatures [32]. The present N,Fe-CDs are more active at elevated temperatures, which matches well the brownstock washing conditions, optimally 62~68 °C in a pulp mill [33]. On the other hand, according to the Arrhenius equation (Equation (7)), the activation energy (E_a_) of the process was calculated to be 33.77 kJ/mol.

(7) $$k={\mathrm{Ae}}^{\frac{-E_{a}}{\mathrm{RT}}}\to \ln\frac{k_{2}}{k_{1}}=\frac{-E_{a}}{R}\left(\frac{1}{T_{2}}-\frac{1}{T_{1}}\right)$$

where E_a_ is the activation energy, kJ/mol; k is the apparent reaction constant, 1/h; A is the Arrhenius constant, 1/h; T is temperature, K; and R is the gas constant, 8.314 kJ/mol/K.

The constant k was linearly correlated with the concentrations of S^2−^ (C_0_) and N,Fe-CDs (C_N_) according to the data measured under varied C_0_ and C_N_ conditions (Figure 4c). Therefore, Equation (1) could be rewritten as Equations (8) and (9). Data in Figure 4c were re-arranged by graphing ln(C_0_/C_A_)/(C_0_C_N_) vs. t according to Equation (9). The linear relationship (R^2^ = 0.9611) was valid as shown in Figure 4d.


(8) $$\ln\frac{C_{0}}{C_{A}}=C_{0}\cdot C_{N}\cdot \;k^{\prime }t$$

(9) $$\frac{1}{C_{0}\cdot C_{N}}\ln\frac{C_{0}}{C_{A}}=\;k^{\prime }t$$

(10) $$\mathrm{where},\;k=C_{0}C_{N}k^{\prime }$$

**Figure 5:**
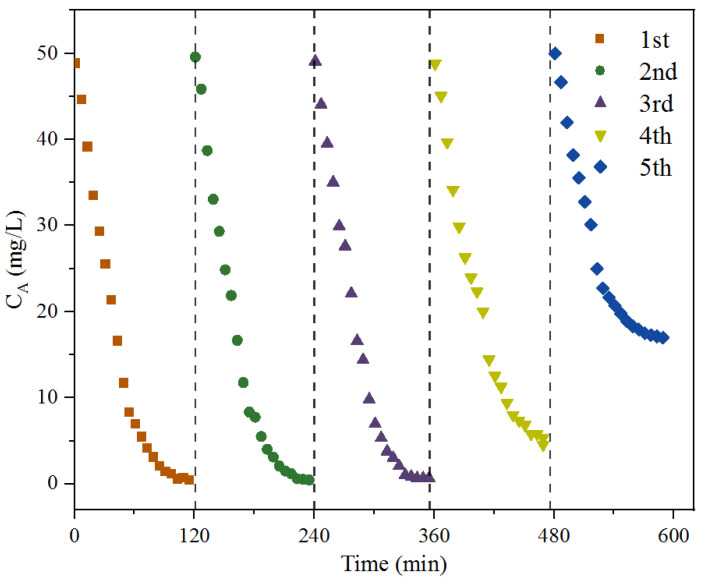
Recyclability of N,Fe-CDs for remediation of S^2−^.

Recyclability is a crucial parameter of a catalyst for practical application in industrial processes. The recycling of the N,Fe-CDs was performed by encapsulating the suspension of the catalysts in a dialysis tubing. In the first run, the tubing containing 300 mg/L of the N,Fe-CDs was immersed with fresh Na_2_S solution. Catalytic oxidation was carried out under conditions of DO of 6 mg/L, an LED light density of 2000 lux, a temperature of 60 °C and duration time of 120 min. The spent N,Fe-CDs in dialysis tubing were re-immersed in fresh Na_2_S solution in the next run. Results in Figure 5 show that a high photocatalytic activity towards S^2−^ (removal ratio > 90%) was maintained after being recycled four times. The catalytic activity was not changed in the first three cycles. In the fourth run, the activity of the N,Fe-CDs decreased slightly but still removed over 92% of the S^2−^. The efficiency of catalysis with the N,Fe-CDs decreased considerably in the fifth cycle, giving a S^2−^ removal of only 66% (Figure 5). The loss of activity of the N,Fe-CDs is possibly because of the S^2−^ oxidation products, e.g., elemental S, adsorbed on surface, reducing the accessibility of the catalyst to free S^2−^. Therefore, dialysis against water for the desorption of S is necessary for the multiple recovery of the catalyst. Similar to conventional photocatalysts such as TiO_2_, N,Fe-CDs can be recycled more than four times, suggesting its good practical performance as a photocatalyst for industrial applications [34]. Good recyclability of CDs has been reported earlier by Hu et al. In their paper, the CDs catalysts also showed outstanding recyclability, being used for six cycles without a substantial loss in catalytic activity [35].

### 3.3. Remediation of KWE by N,Fe-CDs

**Figure 6:**
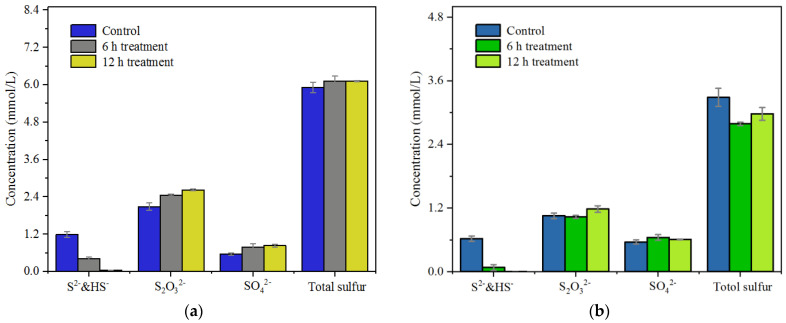
Remediation of S^2−^ in KWE by N,Fe-CDs, (**a**) Original KWE, pH 12.7 and (**b**) Neutralized KWE, pH 7.

Soluble S-containing inorganic compounds in the KWE were mainly S^2−^ (including HS^−^), SO_4_^2−^ and S_2_O_3_^2−^, according to the measurement data (Figure 6). The dissociation of S^2−^ into HS^−^ is majorly because of the strong alkaline pH (12.7) in the KWE. No redox reaction occurs, only dissociation. Both S^2−^ and HS^−^ could be fairly oxidized with our catalyst and quantified by titration analysis. Thereafter, they were taken into calculation together to simplify stoichiometry. After treatment with the N,Fe-CDs for 12 h, the concentration of S^2−^ in the KWE (pH 12.7) decreased from 1.19 to 0.04 mmol/L. The concentration of S_2_O_3_^2−^ increased from 2.09 to 2.63 mmol/L (Figure 6a). In other words, 93.9% of S^2−^ was oxidized to become S_2_O_3_^2−^. Interestingly, the concentration of SO_4_^2−^ increased from 0.56 to 0.84 mmol/L. In terms of total sulfur concentration, there was no difference before and after the catalytic oxidation with the N,Fe-CDs. This suggests no other form of S, such as elemental sulfur, was considerably generated in the catalytic treatment.

The removal of S^2−^ in the neutralized KWE by the N,Fe-CDs is demonstrated in Figure 6b. About half of the S^2−^ in original the KWE was lost after neutralization and the rest was still dissolved in the liquor. Photocatalytic treatment with the N,Fe-CDs removed almost all the free S^2−^ in the neutralized KWE which was decreased from 0.62 to 0.004 mmol/L. The content of S_2_O_3_^2−^ was increased from 1.05 to 1.18 mmol/L. The concentration of SO_4_^2−^ kept relatively stable and total sulfur was slightly reduced by 0.31 mmol/L. There was 42% of converted S^2−^ not detected. A major reason is that the H_2_S generated from the neutralization of the KWE slowly volatilized into the air phase. Another possible explanation is that a part of the S^2−^ was converted into elemental sulfur but not detected in the work. In a pulp mill, there is no pH adjustment in the pulp washing process. It is suggested to perform the catalytic oxidation in the KWE storing tank. The catalyst is also applicable in ETS. The risks of releasing H_2_S or S due to neutralization still need further investigation.

**Figure 7:**
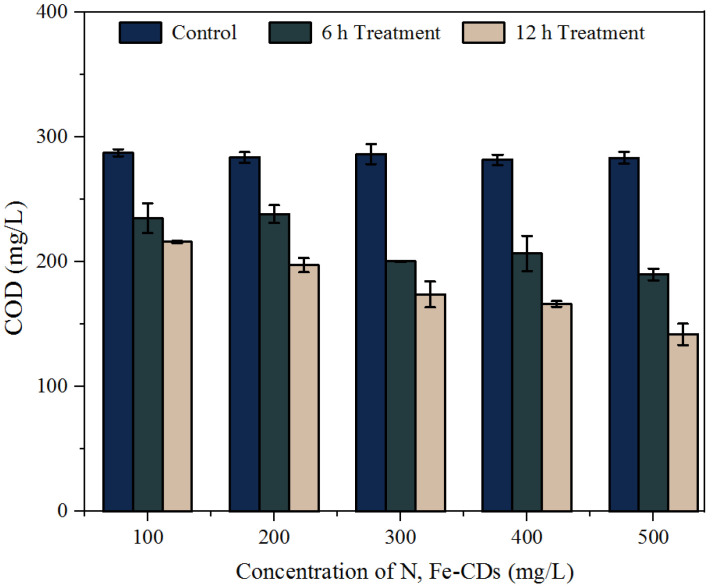
Effects of treatment with N,Fe-CDs on COD of KWE.

The effect of treatment with N,Fe-CDs on the COD of KWE is illustrated in Figure 7. The addition of the N,Fe-CDs up to 500 mg/L did not contribute to the COD. It suggests that citric acid and L-glutamic acid have undergone sufficient oxidation during the hydrothermal fabrication process. After treatment with 500 mg/L N,Fe-CDs for 12 h, the total COD of the KWE was decreased by about 50%. Obviously, there are other reductive substances in the KWE, in addition to S^2−^ and S_2_O_3_^2−^, that could be oxidized by the N,Fe-CDs.

Previously, Selvaraj et al. [36] have reported that electro oxidation process aided with UV-light remediated 100% of sulfide, 92% of COD and 70% of the total organic carbon in tannery effluents. Since N,Fe-CDs are charged nanoparticles, coupling with electro oxidation may further enhance the catalytic capacity of N,Fe-CDs. We and Ye [37] fabricated cyclometalated Ir–Zr metal–organic frameworks as a recyclable visible-light photocatalyst. This novel catalyst could convert sulfide into sulfoxide in water. It inspires us that well-designed catalysts enable direct conversion of sulfide into value-added products through photocatalysis.

It is worth noting that advanced analytical tools such as liquid chromatography-mass spectrometry (LC-MS) are powerful tools for examining the intermediates generated from the photodegradation process [38]. The characterization of S-containing intermediates may help better understand the mechanism of sulfide oxidation by N,Fe-CDs.

### 3.4. Safety Assessment

**Figure 8:**
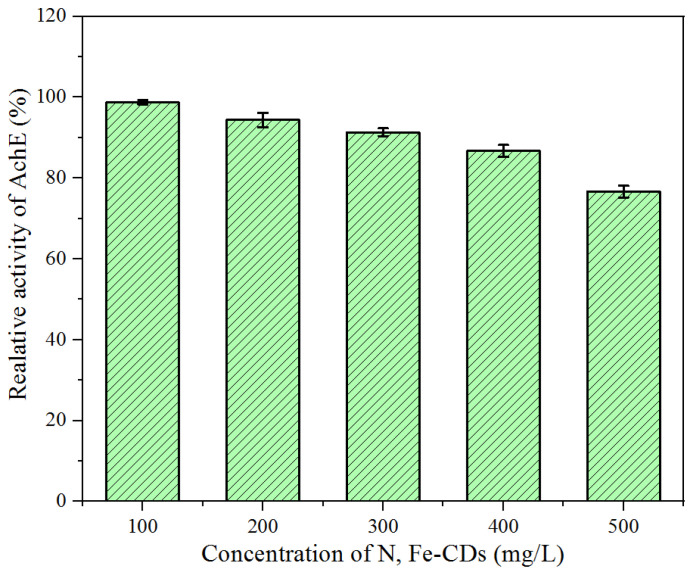
Effect of N,Fe-CDs on the activity of AchE.

AchE is a key enzyme in the animal nervous system. The inhibition of AchE is often measured for the determination of the potential neurotoxicity of drinking water after being contaminated with pesticides, detergents and other bioactive chemicals [39]. The discharging of N,Fe-CDs may impact the safety of a water ecosystem. Therefore, we detected the inhibitory effects of N,Fe-CDs on AchE to evaluate their toxicity. Data in Figure 8 show, in presence of less than 300 mg/L of the N,Fe-CDs, over 90% of AchE activity was preserved. Even when the concentration of the N,Fe-CDs reached 500 mg/L, the loss of AchE activity was around 22%, much lower than 50%, a toxic threshold level. Obviously, our N,Fe-CDs catalyst is safe according to the above assessment. Previously, Suner et al. reported that CDs based on citric acid and arginine had no effect on the activity of AchE at concentrations below 1000 mg/L [40]. However, doping of Ag and Cu in CDs presented strong inhibition. The activity of AchE was decreased by over 60% in the presence of 500 mg/L of Arg-Ag-CDs [40]. By comparison with our data in Figure 8, it can be concluded that Fe-doped CDs have better biosafety.

## 4. Conclusions

The N,Fe-CDs presented in this work were fluorescent nanoparticles (average size 3.18 nm) and had a maximum emission wavelength of 410 nm when excited at 340 nm. The catalytic oxidation of S^2−^ by the N,Fe-CDs followed the first-order kinetic model with an activation energy of 33.77 kJ/mol. The N,Fe-CDs had a good recyclability. A high photocatalytic activity (removal ratio > 90%) towards S^2−^ was maintained after being recycled four times. N,Fe-CDs could efficiently remove S^2−^ in kraft washing effluent (KWE), either in original alkaline or neutralized solutions. After the catalytic remediation of S^2−^, the COD of the KWE was also decreased. The N,Fe-CDs were safe below a content of 300 mg/L according to acetylcholinesterase inhibition tests. The use of N,Fe-CDs may help develop cleaner production processes for kraft brownstock washing. Since odorous S-containing organic compounds such as CH_3_SCH_3_ or CH_3_SSCH_3_ are generated during kraft pulping, the N,Fe-CDs could be further studied to catalytically remove these harmful compounds.

## Data Availability

Not applicable.

## Supplementary Materials

The following supporting information can be downloaded at: https://www.mdpi.com/article/10.3390/polym15030679/s1, Figure S1: Automatic sampling and measurement device for photocatalytic oxidation of S^2−^; Figure S2: Effects of S^2−^ on fluorescence intensity of N,Fe-CDs; Figure S3: High-resolution XPS spectra of N,Fe-CDs.
