# Supplementary material for: Removal of Sulfide Ions from Kraft Washing Effluents by Photocatalysis with N and Fe Codoped Carbon Dots
Source: Polymers (Basel). 2023 Jan 29;15(3):679. doi: 10.3390/polym15030679 (PMC9921700; doi:10.3390/polym15030679)
Supplement: Supplementary file 1 [file polymers-15-00679-s001.zip › polymers-2145337-supplementary.pdf]

Removal of Sulfide Ions from Kraft Washing Effluents by Photocatalysis with N and Fe Codoped Carbon Dots

Hao Luo [1], Hao Liu [1*], and Chengwu Sun [2]

1. State Key Laboratory of Pulp and Paper Engineering, South China University of Technology (SCUT), Guangzhou, Guangdong 510640, China

2. Bengbu-SCUT Research Center for Advanced Manufacturing of Biomaterials, Bengbu 233010, China

List of Supplemental Figures

Figure S1 Automatic sampling and measurement device for photocatalytic oxidation of S^{2-}

Figure S2 Effects of S^{2-} on fluorescence intensity of N,Fe-CDs

Figure S3 High-resolution XPS spectra of N,Fe-CDs

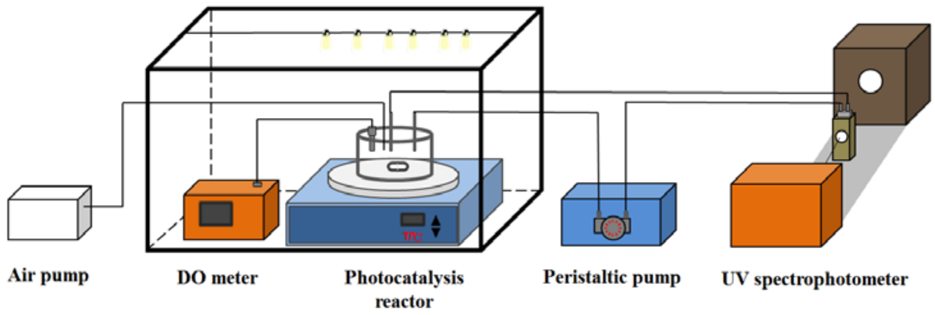

Figure S1 Automatic sampling and measurement device for photocatalytic oxidation of S^{2-}

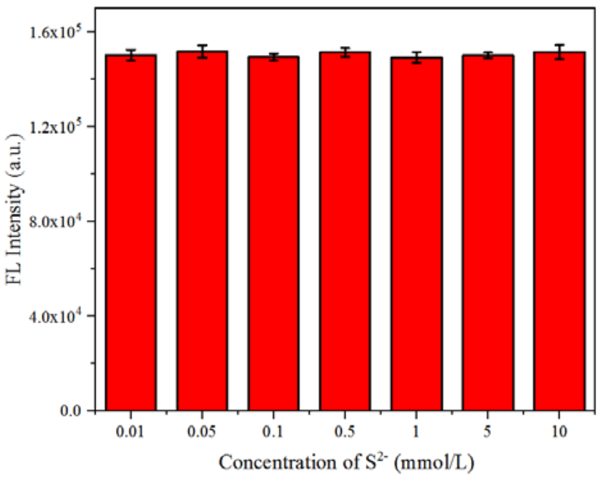

Figure S2 Effects of S^{2-} on fluorescence intensity of N,Fe-CDs

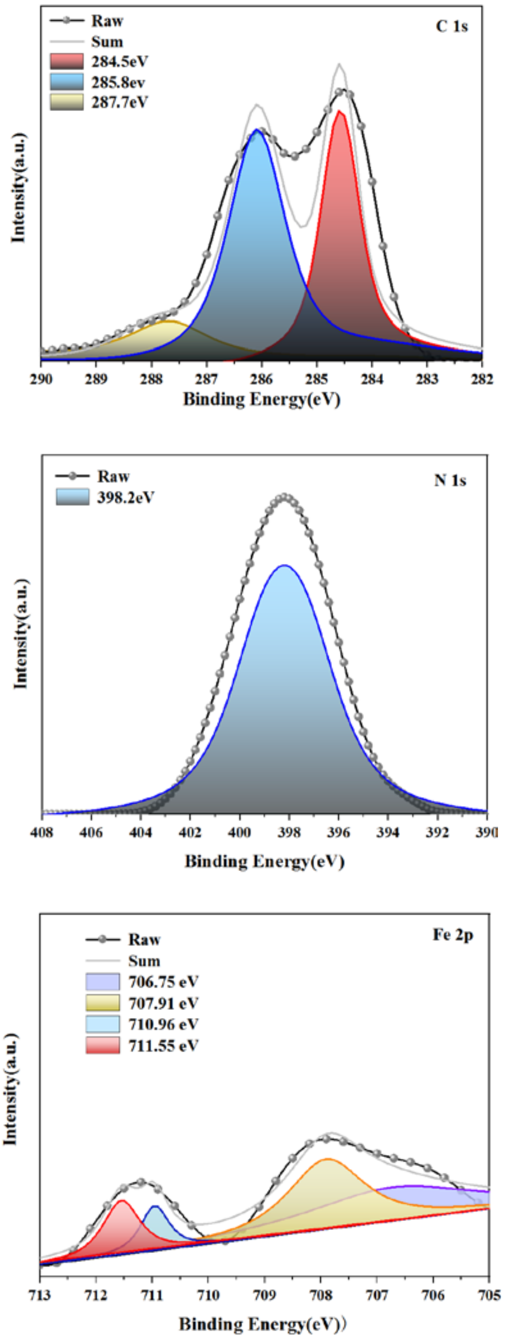

Figure S3 High-resolution XPS spectra of N,Fe-CDs
